# Supplementary material for: Neo-Adjuvant Chemotherapy in Gastric Adenocarcinoma: Impact on Surgical and Oncological Outcomes in a Western Referral Center
Source: Cancers (Basel). 2025 Jul 25;17(15):2465. doi: 10.3390/cancers17152465 (PMC12345789; doi:10.3390/cancers17152465)
Supplement: Supplementary file 1 [file cancers-17-02465-s001.zip › cancers-3716232-supplementary.pdf]

**Table S1. Neoadjuvant protocols employed**	
**Protocols,** *n (%)*	n=110
**Flot**	74(67.3)
**Folfox**	15(13.6)
**Xelox**	1 (0.9)
**ECF**	2(1.8)
**Folfiri**	1 (0.9)
**Cross**	5(4.5)
**Other**	12(10.9)
